# Decitabine combined with CAG regimen in the treatment of elderly patients with acute myeloid leukemia

**DOI:** 10.12669/pjms.36.2.850

**Published:** 2020

**Authors:** Haitao Zhao, Chunyan Wang, Fengying Yu, Qingwei Guo

**Affiliations:** 1 Haitao Zhao, Department of Hematology, Binzhou People’s Hospital, Shandong 256610, China; 2 Chunyan Wang, Department of Hematology, Binzhou People’s Hospital, Shandong 256610, China; 3 Fengying Yu, Department of Pharmaceutical, Binzhou People’s Hospital, Shandong 256610, China; 4 Qingwei Guo, Department of Hematology, Qilu Children’s Hospital of Shandong University, Shandong 250022, China

**Keywords:** Acute myeloid leukemia, CAG regimen, Decitabine, Elderly patients

## Abstract

**Objective:**

To analyze the efficacy and safety of decitabine combined with CAG ((cytarabine + aclacinomycin + granulocyte colony stimulating factor)) regimen and CAG regimen alone in the treatment of elderly acute myeloid leukemia.

**Methods:**

96 elderly patients with acute myeloid leukemia who were admitted to our hospital from July 2015 to July 2017 were randomly divided into an observation group and a control group, 48 cases in each group. The patients in the control group were treated with CAG regimen, while the patients in the observation group were treated with decitabine on the basis of the control group. The clinical curative effect, changes of immune indicators, occurrence of adverse reactions and survival rate at different time after treatment were compared between the two groups.

**Results:**

The total effective rate of the observation group was significantly higher than that of the control group (P<0.05). After treatment, the indicators of cellular immunity in the two groups were significantly lower than those before treatment, and the indicators of cellular immunity in the observation group were significantly lower than those in the control group (P<0.05). There was no significant difference in the incidence of adverse reactions between the two groups (P>0.05). The 9-month survival rate and 1-year survival rate in the observation group were significantly higher than those in the control group (P<0.05).

**Conclusion:**

The combination of decitabine and CAG regimen is effective in the treatment of elderly patients with acute myeloid leukemia. The therapy can fully inhibit cellular immune function and improve long-term survival rate, and its safety has a small difference with that of CAG regimen alone. It is worth clinical promotion.

## INTRODUCTION

Acute myeloid leukemia, one of the most common acute leukemia in clinic, has characteristics of high heterogeneity and invasiveness. According to relevant data, the incidence of acute myeloid leukemia in people over 65 years old is 17.5/100,000, and its incidence is increasing with the aggravation of social aging.^1^,^2^ The main characteristics of acute myeloid leukemia are abnormal proliferation of primitive and immature myeloid cells in bone marrow and peripheral blood. The main clinical manifestations of acute myeloid leukemia are hemorrhage, anemia, infection, fever, abnormal metabolism and organ infiltration. Most patients with acute myeloid leukemia are in serious condition and have a dangerous prognosis and high mortality rate.^3^,^4^ CAG regimen (cytarabine + aclacinomycin + granulocyte colony stimulating factor) is the main chemotherapeutic regimen for acute myeloid leukemia, which has been widely used in clinic, but its effect is not very satisfactory, which is mainly because of the weak body function, poor tolerance and decline of hematopoietic function of bone marrow in elderly patients.^5^,^6^

Decitabine has the function of demethylation and can activate genes which are inactive due to excessive methylation to promote cancer cells to differentiate and die.^7^,^8^ Overseas, the efficacy of decitabine in the treatment of myelodysplastic syndrome has been recognized.^9^ The combination of CAG regimen and decitabine has gradually been applied in clinical practice, which 2has a certain control effect on clinical symptoms and abnormal signs. However, its safety and long-term survival need to be further explored.^10^,^11^

In this study, 96 elderly patients with acute myeloid leukemia in our hospital were selected as the research subjects, and the efficacy and safety of the combination of dicetabine and CAG were analyzed. The purpose of this study was to provide an evidence-based evidence for the improvement of the treatment effect of elderly acute myeloid leukemia.

## METHODS

Ninety-six elderly patients with acute myeloid leukemia who were admitted to our hospital from July 2015 to July 2017 were selected. Inclusive criteria included being diagnosed by bone marrow, chromosome and haematological test, meeting the diagnostic criteria of acute myeloid leukemia in the Standards for Diagnosis and Therapeutic Effect of Hematological Diseases^11^, aged over 60 years old, receiving no other chemotherapy before admission, and having signed the informed consent. Exclusive criteria included having diseases in vital organs, having endocrine diseases, having poor tolerance due to allergic constitution and malnutrition, or having psychiatric diseases. All patients were divided into an observation group and a control group according to random number table method, 48 cases in each group. In the observation group, there were 28 males and 20 females, aged from 60 to 87 years (average 71.5±5.6 years); as to FAB classification, there were 16 cases of M2, 12 cases of M4, 10 cases of M5 and 10 cases of M6. In the control group, there were 25 males and 23 females, aged from 60 to 88 years (average 71.9±5.5 years); as to FAB classification, there were 14 cases of M2, 14 cases of M4, 11 cases of M5 and 9 cases of M6. The results could be compared between the two groups as there was no significant difference in general data (P>0.05).

Patients in the control group were treated with CAG regimen alone. Firstly, they were intravenously infused with 20 mg of aclacinomycin (Guangzhou Yipinghong Pharmaceutical Co., Ltd., China; approval number: H20060159) and 250 mL of 5% glucose injection from the 1^st^ day of chemotherapy to the 8^th^ day, once a day. Aclacinomycin continued to be used in the first and second week after chemotherapy. Cytarabine (Harbin Laibotongn Pharmaceutical Co., Ltd., China; approval number: H23021806) was intravenously infused at a dose of 2 mg/kg at the 1^st^ day~14^th^ day of chemotherapy, once a day. At the day after the first treatment course, granulocyte colony-stimulating factor (Kyowa Kirin Joint-stock Company; approval number: S200100631) was subcutaneously injected once at the dosage of 200 μg/m^2^ if the patients had no severe bone marrow suppression and juvenile cells in peripheral blood. Granulocyte colony-stimulating factor was not used if there was no bone marrow suppression. Symptomatic treatment such as liver protection, vomit stopping and nutritional support was given during chemotherapy. If the level of hemoglobin was lower than 60 g/L, erythrocyte suspension was used; when the platelet was lower than 20×10^9^/L, thrombopoietin and single donor platelets were injected.

On the basis of the treatment of the control group, the observation group was treated with ceftabine (Jiangsu Hausen Pharmaceutical Co., Ltd., China; approval number: H20130067) at the dosage of 15 mg/m^2^, at the first to fifth day of chemotherapy. From the first day to the fifth day of chemotherapy, ceftabine was mixed with 250 mL of 0.9% sodium chloride injection, and the mixture was intravenously infused. Twenty days was regarded as one treatment course, and they were treated for 60 days.

The changes of CD4^+^, CD3^+^, CD8^+^T cells in the two groups before and after treatment were detected by a fully automatic flow cytometry and its associated reagents (Partec Company, Germany; model type: CyFlow). The occurrence of adverse reactions in the two groups was recorded, including bone marrow suppression, gastrointestinal reaction, secondary infection and liver function damage. They were followed up for one year after treatment, and the three-month, nine-month and one-year survival rates were compared between the two groups.

The clinical efficacy of the two groups was evaluated according to the Standards for Diagnosis and Therapeutic Effect of Hematological Diseases.^3^ Complete remissions was determined if clinical symptoms disappeared, hemogram was normal, the percentage of immature cells in bone marrow was less than 5%, bone marrow increase was basically normal. Partial remission was determined if clinical symptoms improved, hemogram was partly normal, and the percentage of immature cells in bone marrow was 5%-20%. No improvement was determined if clinical symptoms remained unchanged or aggravated, hemogram indicators were still positive, and the percentage of immature cells in bone marrow was more than 20%. Total effective rate could be calculated using the formula: total effective rate = (number of cases of complete remission + number of cases of partial remission)/total number of cases×100%.

### Statistical analysis:

Data were processed by SPSS 21.0, and measurement data were expressed by mean ± standard deviation and processed by t-test. Count data was expressed by rate (%) and processed by Chi-square test. Difference was considered as statistically significant if P<0.05.

## RESULTS

The total effective rate of the observation group was 79.17% and that of the control group was 54.17%. The total effective rate of the observation group was higher than that of the control group, with statistical significance (P<0.05, Table-I).

**Table-I:** Clinical efficacy between two groups.

Group	Observation group	Control group	X^2^	P
Complete remission	11(22.92)	5(10.42)		
Partial remission	27(56.25)	21(43.75)		
No improvement	10(20.83)	22(45.83)		
Total effective rate	38(79.17)	26(54.17)	5.071	<0.05

There was no significant difference in cellular immune indicators between the two groups before treatment (P>0.05). The cellular immune indicators of the two groups after treatment were significantly lower than those before treatment (P<0.05). The indicators of the observation group were significantly lower than those of the control group after treatment (P<0.05); the differences were statistically significant (P<0.05, Table-II).

**Table-II:** Cellular immune indicators between two groups.

Group		Observation group	Control group
CD3+T cell	Before treatment	54.37±6.66	54.31±6.64
	After treatment	40.02±3.48*^#^	46.37±4.61*
CD4+T cell	Before treatment	36.81±5.60	36.85±5.61
	After treatment	27.95±4.28*^#^	32.08±4.37*
CD8+T cell	Before treatment	28.78±5.74	28.65±4.95
	After treatment	21.94±4.39*^#^	25.16±4.37*

* indicated P<0.05 compared to before treatment in the same group,

# indicated P<0.05 compared to the control group.

Nineteen patients in the observation group had adverse reactions, including 5 cases of renal insufficiency, 6 cases of nausea and vomiting, 5 cases of alopecia and 3 cases of pulmonary infection. Sixteen patients in the control group had adverse reactions, including 4 cases of renal insufficiency, 5 cases of nausea and vomiting, 4 cases of alopecia and 3 cases of pulmonary infection. The incidence of adverse reaction of the observation group was 39.58%, and that of the control group was 33.33%; there was no significant difference between the two groups (X^2^=0.726, P>0.05).

The nine-month survival rate and one-year survival rates in the observation group were significantly higher than those in the control group (P<0.05, Table-III).

**Table-III:** Survival rate between two groups at different time points after treatment.

Group	Observation group	Control group	X^2^	P
After three months	44(91.67)	42(87.50)	0.568	>0.05
After nine months	29(60.42)	18(37.50)	4.052	<0.05
After one year	17(35.42)	4(8.33)	9.041	<0.05

## DISCUSSION

Acute myeloid leukemia is a common malignant hematological malignant tumor, and it usually occurs to the elderly.^12^ In recent years, the incidence of acute myeloid leukemia is on the rise. The exact etiology and specific mechanism of acute myeloid leukemia have not yet been fully clarified.^13^ However, elderly patients often have poor physiological function and organ function, and they usually have one or more chronic diseases, poor tolerance and low sensitivity to antineoplastic drugs; hence clinical treatment for them is difficult, which also forces clinicians to explore new alternative treatment methods.^14^

CAG regimen is a common regimen for the treatment of acute myeloid leukemia.^15^ Aclacinomycin, cytarabine and granulocyte colony stimulating factor were used for chemotherapy in CAG regimen. Aclacinomycin is an anthracycline antineoplastic drug with strong lipophilicity and high-speed operation. Moreover, aclacinomycin exists in cells with a high drug concentration. Cell cycle is blocked by embedding DNA of pathological cells and inhibiting the formation of nucleic acid.^16^ Cytarabine, a specific drug for cell cycle, mainly acts on cells in S proliferation phase and can kill tumor cells by inhibiting the DNA synthesis of cells and accelerate the proliferation of interfering cells.^17^ Granulocyte colony stimulating factor is a glycoprotein that can directly act on hematopoietic progenitor cells to accelerate differentiation and proliferation, promote the maturation of granulocytes and mononuclear macrophages as soon as possible, enhance the functions of eosinophils and macrophages, kill leukemia cells and enhance the sensitivity of immature cells to drugs.^18^

In recent years, with the deepening of clinical research on hematological malignancies, it has been found that the formation of acute myeloid leukemia is related to the abnormal methylation of anticancer genes such as HIC-1A, P15 and ER in patients.^19^ When the abnormal methylation of anti-cancer genes occurs, it will weaken the anti-cancer ability of anti-cancer genes, leading to further development and differentiation of tumors, and affect the role of chemotherapeutic drugs. Therefore, the efficacy of CAG regimen alone in the treatment of elderly patients with acute myeloid leukemia is not good. This study was based on the traditional CAG regimen plus dexamethabine treatment. Dicetabine can inhibit the proliferation of cancer cells by inhibiting DNA methyltransferase and reducing DNA methylation and moreover can avoid the occurrence of drug resistance, which is the strongest specific inhibitor of DNA methylation.^20^ The results of this study showed that the total effective rate of the observation group was significantly higher than that of the control group, which was consistent with a previous study.^21^ The mechanism of decitabine enhancing the efficacy of CAG regimen might be that decitabine is a kind of demethylation drug, and its main active ingredient is a deoxycytidine analogue, which can inhibit cytotoxicity in high concentration and promote cell differentiation in low concentration and has high anti-tumor activity. In addition, decitabine combined with CAG regimen can improve the cytotoxicity of adenosine arabinate. Studies have indicated that the cellular immune indicators decreased significantly after treatment,^22^,^23^ while the level of peripheral blood regulatory cells increased significantly, indicating that patients with acute myeloid leukemia are in the state of immunosuppression. Chemotherapy for patients with acute myeloid leukemia can effectively kill leukemic cells, but it can also cause damages of immune cells.

This study found that the cellular immune indicators after treatment were significantly lower than those before treatment in both group and the indicators in the observation group were significantly lower than those in the control group after treatment, which was consistent with Messingerova et al.^24^ Those results suggested that decitabine combined with CAG regimen could cause the weakening of immune function in elderly patients with acute Myelocytic Leukemia compared with CAG regimen alone. The reason for the above phenomenon might be that decitabine could establish a stable regulatory T cell line through inducing the significant increase of Foxp3-mRNA expression level and induce CD4^+^CD25^+^T cells to differentiate into CD4^+^CD25^+^regulatory T cells.^25^ Moreover, this study also found that there was no significant difference in the incidence of adverse reactions between the two groups (P>0.05), suggesting that the combination of decitabine and CAG regimen would not increase the incidence of adverse reactions in elderly patients. The nine-month and one-year survival rates in the observation group were significantly higher than those in the control group, suggesting that the combination of decitabine and CAG regimen could significantly improve the long-term survival rate in elderly patients with acute myeloid leukemia.

## CONCLUSION

The combined use of decitabine and conventional CAG regimen in elderly patients with acute myeloid leukemia can enhance the blocking effect on cellular immune function, which can not only significantly improve the short-term efficacy, but also enhance the long-term survival rate, without increasing the risk of adverse reactions. The therapy is worth promotion.

### Authors’ Contribution:

**HTZ & QWG:** Study design, data collection and analysis.

**CYW, FYY & QWG:** Manuscript preparation, drafting and revising the manuscript.

**HTZ & QWG:** Review and final approval of manuscript.
